# siRNA-mediated stathmin1 silencing inhibits proliferation of prostate carcinoma cell line

**DOI:** 10.55730/1300-0152.2612

**Published:** 2022-05-31

**Authors:** Asude AKSOY, Selcen GÖKTÜRK, Ebru ETEM ÖNALAN, Ahmet TEKTEMUR, Gökhan ARTAŞ, Asuman VAROĞLU, Mustafa KOÇ

**Affiliations:** 1 Department of Medical Oncology, Medical Faculty, Firat University, Elazig, Turkey; 2 Department of Internal Medicine, Medical Faculty, Firat University, Elazig, Turkey; 3 Department of Medical Biology and Genetics, Elazig, Turkey; 4 Department of Pathology, Medical Faculty, Firat University, Elazig, Turkey; 5 Department of Neurology, Medical Faculty, Medeniyet University, Istanbul, Turkey; 6 Department of Radiology, Medical Faculty, Firat University, Elazig, Turkey

**Keywords:** Prostate cancer, Stathmin1, siRNA silencing, autophagy, invasion, apoptosis

## Abstract

Stathmin1 (STMN1) has been proposed as a possible prognostic marker and a potential therapeutic target for some cancers. We aimed to analyze the changes in autophagy, invasion, apoptosis-related genes in prostate cancer (PCa) cell line (PC-3), after small interfering RNA (siRNA)-mediated STMN1 silencing, and also the relationships of STMN1 expression, clinicopathological parameters, and survival (OS) in PCa cases.

The STMN1 expressions were analyzed, immunohistochemically, in formalin-fixed paraffin-embedded 75 PCa and 15 benign prostatic hypertrophy (BPH) tissues. The correlation between the levels of expression STMN1, clinicopathological features, and OS was determined in PCa cases. The siRNA-mediated STMN1 incubated PC-3 cells were transfected and compared to negative control siRNAs. We determined mRNA levels in autophagy, invasion, and apoptosis genes with the combination of reverse transcription-polymerase chain reaction (RT-PCR) and western blotting in PC3 cell lines after STMN1 silencing.

It was determined that STMN1 was overexpressed significantly in PCa cases, immunohistochemically. The overexpression of STMN1 was significantly correlated with the high-grade Gleason score, and it was associated with a worse prognosis of PCa cases according to the Kaplan–Meier survival analysis (p < 0.05). Significant silencing in STMN1 was determined (87.5%) after siRNA applications. Especially, invasion genes such as claudin 7, fibroblast growth factor 8, hypoxia-inducible factor 1 subunit alpha, hepatocyte growth factor, matrix metallopeptidase 2, 7 genes, markedly, decreased by siRNA-mediated STMN1silencing. STMN1 silencing was determined to significantly increase caspase 3 protein expression by using western blot analysis (p < 0.001). Although STMN1 silencing did not have a significant effect on the induction of apoptosis and autophagy-related genes in PCa cells, it was shown to affect apoptotic mechanisms through the caps3 protein.

siRNA-mediated STMN1 silencing decreases proliferation in the PCa cell line. It is thought that STMN1 can serve as a potential therapeutic target in the advanced stage-PCa, especially.

## 1. Introduction

Prostate cancer (PCa) is one of the most common malignancies in men with hormonal factors that play a role in the pathology and is generally a disease of the elderly man (Siegel et al., 2020). PCa is known to be 10.6% of all cancers, in 2020. At the time of diagnosis, 76% of the cases are in the locally advanced stage, 13% with regional lymph nodal involvement, and 6% distant metastasis. The recurrence develops in about 40%–50% after first local (i.e. prostatectomy or radiation) treatments in the locally advanced stage. Although androgen deprivation therapy (ADT) is a cornerstone for the management of advanced PCa, sensitivity to ADT will determine the course of the disease (Vlajnic et al., 2021). The androgen receptor (AR) signaling becomes resistant to ADT within a few years; cytotoxic therapies are also included in the management of the advanced stage of PCa. ‘High-risk’ PCa often progresses to castration-resistant PCa. The 5-year survival of PCa in advanced stages is about 30.2%. Microtubule-targeting agents, such as taxanes, are the first option in treatments of advanced stage PCa. Without microtubule growth and assembly, the mitotic spindle cannot form and the cell cycle ceases. Taxanes decrease depolymerization of β-tubulin during the mitotic cycle, resulting in mitotic arrest and following cell death (NCCN, 2021). Taxanes kill prostate cancer cells through this mechanism and prevent proliferation in PCa.

Some signaling pathways such as phosphoinositide 3-kinase (PI3K) and hedgehog are activated during the metastatic process and resistant to drugs in the treatment of PCa. In these pathways, cyclin-dependent kinases (CDKs), mitogen-activated protein kinases (MAPKs), phosphoinositide 3-kinase (PIK3), protein kinase A (PKA), c-Jun N-terminal kinase (JNK), and Ca^+2^/calmodulin-dependent protein kinases (CamKs)/phosphate signaling pathways, are regulated by stathmin1 (STMN1) (Murillo-Garzón et al., 2017). STMN1, also known as an oncoprotein, is an important cytoplasmic phosphoprotein that regulates cellular microtubule dynamics. STMN1 stimulates microtubule depolymerization by sequestering tubulin and inducing catastrophes (Howell et al., 1999; Yan et al., 2018). In addition, STMN1 has a role in the cell signaling pathway. STMN1 contributes to a malignant phenotype characterized by a high proliferative index (Ghosh et al., 2007). High STMN1 expression has been associated with poor prognosis in many different tissue-derived cancer types such as hepatoma, cholangiocarcinoma (Watanabe et al., 2014). In previous studies, both in vivo and in vitro, it has been shown that the proliferation, invasion rate can be reduced by genetically silencing STMN1 with siRNAs in mesothelioma, gastric cancer, colon cancer cell lines (Kimberly et al., 2015).

Autophagy and energy metabolism are strongly associated with the cell cycle. Abnormal autophagy-apoptosis mechanisms are closely associated with the developments of some cancer and autoimmune diseases. Autophagy functions nearly like a tumor suppressor gene (TSG) in the early stages of tumor genesis. Often, malignancy occurs via the inhibition of autophagy mechanisms. It has been shown that autophagy can be induced in the PCa cell line through the down-regulation of methyltransferases (KMT) and demethylases (KDM), which are important epigenetic histone modifiers (Sha et al., 2020).

The detection of the molecular changes in the invasion, proliferation, formation of PCa would determine the essential goals of the treatment. In the literature, there are no studies investigating autophagy, apoptosis, and invasion mechanisms in the pharmacological inhibition of STMN1, which will increase the effect of microtubule inhibitors used in the treatment of PCa and at the same time, reduce tumor proliferation (Mistry et al., 2005).

STMN1 has been proposed as a possible prognostic marker and a potential therapeutic target for some cancers (Hiıeh et al., 2010, Watanabe et al., 2014). Based on the aforementioned studies, we proposed a hypothesis; STMN1 expression may be a regulatory step in the pathophysiology of the formation and spread of PCa, and the activities of STMN1 can be knockdown by STMN1 silencing with siRNAs. In this study, we investigated whether siRNA-mediated STMN1 silencing in PC3 cell lines affects autophagy, invasion, apoptosis-related gene panels, and whether the expression of STMN1 can be evaluated as a prognostic marker in PCa cases.

## 2. Materials and methods

### 2.1. Case selection

Biopsy materials of 90 patients, 75 of whom were diagnosed with PCa and 15 of whom were diagnosed with benign prostatic hypertrophy (BPH) as the control group, were included in the study. Before the study, Ethics Committee approval was obtained to employ the retrospective medical data of patients that were treated between 2009 and 2017 in our university, medical oncology department. The paraffin blocks of prostate tissues (transurethral resection (TUR) samples) were obtained from the pathology department of the faculty of medicine of our university. Specimens were divided into 6 groups according to the Gleason score (GS) system of WHO 2016; benign prostate hyperplasia (BPH), as control group, grade group 1 (GS ≤ 6), grade group 2 (GS = 7 (3+4)), grade group 3 (GS = 7 (4+3)), grade group 4 (GS = 8), and grade group 5 (GS = 9–10) (NCCN, 2021).

### 2.2. Tissue samples and immune histochemistry

**Figure 1:**
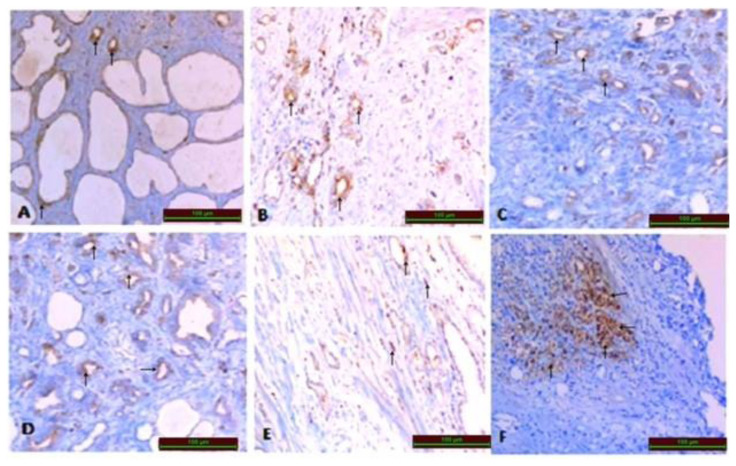
Histopathological evaluation for stathmin1 (STMN1). A) STMN1 (+) expression evaluation in the control group (Haematoxylin & Eosin (HE) X200), Immunoperoxidase X 200); B) STMN1 immunoreactivity in Grade 1 prostate acinar adenocarcinoma group (Haematoxylin & Eosin (HE) X 100), Immunoperoxidase X 100); C) STMN1 immunoreactivity in Grade 2 prostate acinar adenocarcinoma group (Haematoxylin & Eosin (HE) X 100), Immunoperoxidase X 100); D) STMN1 immunoreactivity in Grade 3 prostate acinar adenocarcinoma group (Haematoxylin & Eosin (HE) X 100), Immunoperoxidase X 100); E) STMN1 immunoreactivity in Grade 4 prostate acinar adenocarcinoma group (Haematoxylin & Eosin (HE) X 100), Immunoperoxidase X 100); F) STMN1 immunoreactivity in Grade 5 prostate acinar adenocarcinoma group (Haematoxylin & Eosin (HE) X 100), Immunoperoxidase X 100).

5-μm-thick paraffin block sections of the tissues fixed with routine formalin buffer were transferred to poly-lysine slides. These were processed in an automated staining device STMN1 (Mouse monoclonal Ig G, 1/200, BOSTER/ USA) staining. The preparations were inspected, assessed, and photographed with a Leica DM500 microscope. The histoscores were determined based on the intensity (0.1: <25%, 0.4: 26%–50%, 0.6: 51%–75%, 0.9: 76%–100%) and severity of immune reactivity (0: no, +0.5: very little, +1: little, + 2: medium, +3: severe) (Figures 1A–1D). The histoscores were calculated with the “intensity X severity” formula (Chen et al., 2012). The STMN1 histoscores were categorized into two subgroups: those that were lower and higher than median values.

### 2.3. Cell culture and siRNA transfection

To evaluate the utility of STMN1 gene silencing as a therapeutic target, the effect on apoptotic, invasions, and proliferation-related gene expressions of STMN1 siRNA transfection in PC-3 cell lines was analyzed. Human PC-3 cells (ATCC^®^ CRL-1435^™^) were grown as monolayers in the RPMI-1640 medium (Cat. No. R0883, Sigma-Aldrich, Germany) with 10% fetal bovine serum (FBS; Cat. No. F6178, Sigma-Aldrich, USA), 100 μg/mL^−1^ streptomycin and 40IU/mL^−1^ penicillin. PC-3 cell lines were incubated with 95% air plus 5% CO_2_ at 37° (Nuve, Turkey). For siRNA transfection of PC-3 cells, The HiPerfect^®^ transfection reagent (Cat. No. 301704, Qiagen, Germany) was employed with 5 μg of STMN1-siRNAs (per 35 mm culture dish) (Cat. No. GS3925, Qiagen, USA) or negative control siRNA (Cat. No. 1027280, Qiagen, USA) on 6-well cell culture plates at a state of 70–80 confluency. Transfection was done from 36 to 48 h after transfection. To confirm transfection efficiencies and suppression of STMN1-RNA in PC-3 cells were performed by qRT-PCR (with Cat. No. PPH14448B primers, Qiagen, Germany). qRT-PCR results were calculated as the percentage of siRNA silencing with the ΔΔCT mean = ΔCT STMN1-siRNA - ΔCT negative control; fold change = 2^−ΔΔCT^; percentage of silence = 100 × (1-fold change) formula.

### 2.4. qRT-PCR analysis

Total cellular RNA was isolated from PC-3 cells with the Gene Jet RNA Purification kit (Cat. No. K0731, Thermo Scientific, Lithuania) according to the manufacturer’s protocols. The RNA pellet was resuspended in 10–30 μL nuclease-free water. The RNA samples were reverse transcripted the complementary DNA (cDNA) with a High-Capacity cDNA Reverse Transcription Kit (Cat. No. 4368814, Applied Biosystems, USA) and a thermal cycler (Veriti, Applied Biosystems, Singapore) at 25 °C for 10 min, 37 °C for 120 min, and 85 °C for 5 min. SYBR green-based autophagy (Cat. No. HATPL-I, Human Autophagy Primer Library, RealTimePrimers.com), apoptosis (Cat. No. HPA-I, Human Apoptosis Primer Library, RealTimePrimers.com), and invasion panels (Cat. No. HTIM-I, Human Invasion Primer Library, RealTimePrimers.com) were used for qRT-PCR analysis. The qRT-PCR was conducted on an Applied Biosystem 7500 Fast system (Applied Biosystems, Foster City, CA, USA) using universal SYBR green supermix (Cat. No. 172-5121, Bio-Rad, USA) for 41 genes selected from three panels. Glyceraldehyde 3-phosphate dehydrogenase (GAPDH) (Cat. No. QT00079247, Qiagen, USA) was used as an internal standard. The 2^−ΔΔCT^ method was employed to determine the gene expression differences after the qRT-PCR analysis. We repeated PCR measurements three times, and the reported measurements were based on these triplicate analyses.

### 2.5. Western blotting

Homogenization of the cells was performed on ice 1:10 (w/v) in a buffer containing 50 mM Tris (pH 7.4), 0.1 mM NaCl, 1% Triton X-100, 5 mM EDTA, 1.0 mM phenylmethylsulfonyl fluoride, 10 mg/mL aprotinin and 10 mg/mL leupeptin. The measurement of protein concentration for each sample was performed according to the Lowry procedure using a protein assay kit (Sigma, St. Louis, MO, USA), and western blotting was performed. Separated proteins with SDS polyacrylamide gel electrophoresis were transferred to nitrocellulose membranes (Santa Cruz Biotechnology, Inc., Texas, and USA). Nitrocellulose blots were blocked with 5% dry milk and probed with Caspase 3 (sc-7272, Santa Cruse Biotechnology, Texas, USA) and Lc-3 (sc-398822, Santa cruse) primary antibodies at a dilution of 1:500. The blots were washed and incubated for 1 h with a secondary antibody, antimouse or antirabbit Ig peroxidase-conjugated (Santa Cruz Biotechnology, Inc., Texas, and the USA) at a dilution of 1:500. Specific binding was detected with chemi-glow detection reagents using chemiluminescent detection. The relative amount of immunoreactive bands on Western blots was quantified in arbitrary units by scanning blots using a computerized software program (LabWorks 4.0; UVP, Inc., Cambridge, UK). Primary human anti-Beta actin and secondary antimouse antibodies were used to monitor Beta-actin expression as a loading control.

### 2.6. Statistical analysis

SPSS (IBM corp. Armonk, NY, USA) version 22 was employed in statistical analyses. The collected data were presented as mean ± standard deviation. Chi-squared test statistics were used to compare categorical measurements between groups. The Mann–Whitney U test was used to compare numerical measurements that did not show normal distribution between them. In comparison, the Kruskal–Wallis test was used for the variables between more than two groups. Spearman/Pearson correlation and Student’s t-test were used to determine the parameters affecting the median overall survival (OS). Survival curves were calculated according to the Kaplan–Meier method using Cox regression, and differences between curves were evaluated using the log-rank test. The median OS was calculated from the date of diagnosis to the date of death or last follow-up. ROC analysis was performed to determine the cutoff value for the value of STMN1 expression. The qRT-PCR data were analyzed with the ΔΔCt module at the Qiagen Gene Globe Data Analysis Center portal (http://www.qiagen.com/us/shop/genesand-pathways/data-analysis-center-overview-page/). The qRT-PCR module transformed the threshold cycle (Ct) values to determine gene expressions. The findings in the 95% confidence interval (CI) were considered statistically significant (p < 0.05).

## 3. Results

### 3.1. STMN1 expression in prostate cancer tissue by immunohistochemistry

**Figure 2:**
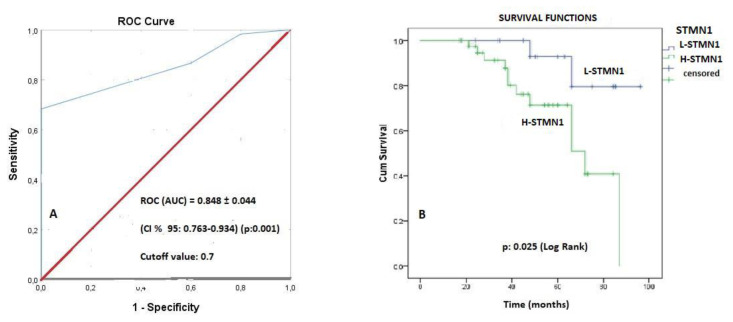
A) The receiver operating characteristic curve (ROC) for stathmin1 (STMN1) expression; B) Kaplan–Meier survival curves stratified by stathmin1 (STMN1) groups.

STMN1 was immune reactive in PCa and BPH tissues as a control group (which is diagnosed as BPH disease). STMN1 overexpression was statistically significantly increased in PCa tissues compared to BPH tissues (p = 0.001) as shown in Figure 1 and Table 1. The general clinical characteristics of the patients are shown in Table 2. The cutoff value of STMN1 in cases’ tissues with PCa was tested with the ROC (Receiver Operation Characteristics) curve. For the cutoff value of STMN1, AUC (area under the curve) = 0.848 ± 0.044 (CI % 95: 0.763–0.934) (p < 0.001) and *0.7* were taken as cutoff value (sensitivity 68% and specificity 100%) as shown in Figure 2A. We defined *< 0.7* CD1 as a low (L-STMN1) group and *≥ 0.*7 CD1 as a high (H-STMN1) group. General clinical features of cases according to STMN1 expression are shown in Table 3.

There was a statistically significant positive correlation between the overexpression of STMN1 and high-grade Gleason score, the value of lactic dehydrogenase enzyme (LDH) (Pearson’s r = 0.488, p < 0.001, Pearson’s r= 0.207, p < 0.05, respectively). A negative correlation was also observed between OS and STMN1 expression (Pearson’s r = −0.420, p = 0.042). No significant correlation was found between STMN1 expression and other parameters (grades, age, pretreatment prostate-specific antigen (PSA) value, last visit-PSA value, stage) are shown in Table 4.

### 3. 2. Survival evaluation

The median follow-up time was 75 (17–96) months, and the median OS was 87 months (CI 95%: 65.45–108.54) in the H-STMN1group. Although it was not reached the median OS value in the L-STMN1 group statistically significant correlation was found between STMN1 groups (H/L) for the median OS (p = 0.025) as shown in Figure 2B.

### 3. 3. siRNA transfection efficiency analysis

**Figure 3:**
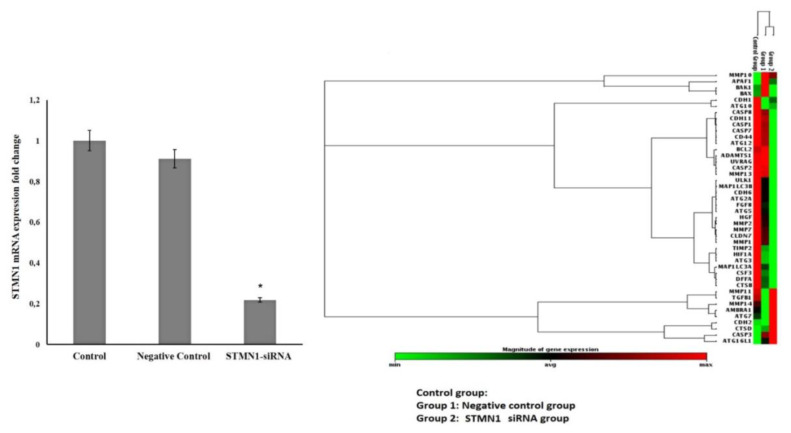
Heat map of the effects on gene expression after the transfection stathmin1 (STMN1) siRNA in PC-3 cell line. The mR NA expression obtained using qPCR for the targets selected from the genes in Table 4 is shown as a graph of the profile of the heat map. Genes clustered according to their expression patterns. The red color on the heat map indicates genes that are highly expressed compared to control; green color, genes that are expressed at a low level, whereas black color represents genes that are equal to the control.

The mRNA expressions of STMN1 in siRNA groups demonstrated a significant decrease when compared to the control group (p = 0.007). The silencing of the STMN1 siRNA rate was *87.5%*, with the gene silencing calculation method for applied biosystems (Figure 3A–B).

A total of 41 genes were successfully analyzed. The STMN1 silencing markedly suppressed the proliferation-invasion related claudin7 *(CLN7)*, fibroblast growth factor8 *(FGF8)*, hypoxia-inducible factor1 *(HIF1A)*, hepatocyte growth factor *(HGF)*, hypoxia-inducible factor 1, alpha subunit *(HIF1A)*, matrix metallopeptidase 2 (gelatinase A) *(MMP2)*, matrix metallopeptidase7 (matrilysin, uterine*) (MMP7);* apoptosis-related genes caspase1 *(CASP1)*, and caspase7 *(CASP7)*, and caspase8 *(CASP8)* and autophagy-related ATG2 autophagy-related two homolog A *(ATG2A), (ATG5)*, Microtubule-associated protein 1 light chain 3 alpha *(MAPILC3A)*, Unc-51-like kinase 1 (C. Elegans) *(ULK1)* gene expressions *(*p < 0.05*)* as shown in Table 5. Interestingly, the STMN1 silencing has only upregulated the expression of cathepsin D (fold change: 5.242).

### 3.4. siRNA-mediated STMN1 silencing induced apoptosis via the caspase 3 pathway

**Figure 4:**
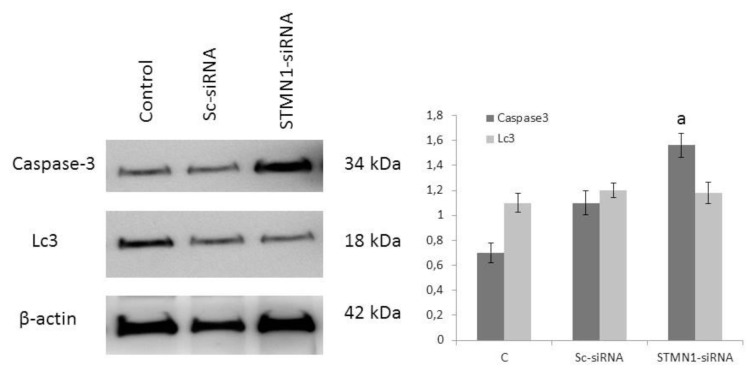
Western blot analysis of caspase 3 and LC-3 proteins in PC-3 cell line. The β-actin was used as an internal control. PC-3 cells were treated with Sc-siRNA and stathmin1 (STMN1)-siRNA for 48 h. The densitometer-intensity data of the protein bands of each blot is represented as mean ± SEM from three independent experiments. ^a^ letter p < 0.05 versus control as measured by one-way ANOVA test.

To determine the possible apoptotic and autophagic mechanism of STMN1 contributing to PCa cells, we examined CASP3 and Lc-3 protein expression by using western blot analysis. The experiment was divided into three groups as transfection reagent (Control; C), scrambled si RNA group (Sc-siRNA), and STMN1 siRNA groups (STMN1-siRNA). Results revealed that *CASP3* was significantly higher in the STMN1-siRNA group than in the control and Sc-SiRNA groups. STMN1 silencing was determined to significantly increase *CASP3* protein expression, but there was no change in LC3 protein expression (p < 0.001 and p = 0.32) as shown in Figure 4. These results suggested that the inhibition of STMN1 could induce the apoptosis of PC3 cells via the caspase3 pathway.

## 4. Discussion

Although direct comparative data are not available to inform treatment decisions, treatment options are very limited in castration resistance metastatic prostate cancer (mCRPCa), and in the advanced stage, only taxanes as cytotoxic therapy have been shown to provide a survival advantage (Hsu et al., 2020). In the treatment of PCa, alternative cytotoxic treatment options enzalutamide and darolutamide are settled in almost every stage of PCa today (Armstrong, 2018). We also thought about why cytotoxic chemotherapies are not so effective and why resistance to taxane treatments is developing in a short time. Starting from here, we aimed to determine the molecular mechanisms in PCa, and whether STMN1 has a place in the carcinogenesis steps, and the results of its inhibition both in vitro and in vivo, in this study. STMN1, a named oncoprotein 18, plays an important role through activation of the PI3K/Akt pathway in many solid tumor metastases. STMN1 acts as a microtubule destabilizer to promote cell proliferation, and also it has a role in the cell signaling pathway. STMN1 has a dual format due to pleiotropic phenomena. STMN1 acts both decreasing cancer invasion and promoting carcinogenesis (Williams et al., 2012).

It is known that STMN1 is intensely expressed in many cancers such as lung cancer, breast, and prostate cancer (Friedrich et al., 1995, Golouh et al., 2008, Nie et al., 2015). It has been reported that overexpression of STMN1 is related to poor prognosis, and resistance to treatments in many cancers (Friedrich et al., 1995, Hiıeh et al., 2010, Watanabe et al., 2014). Pharmacological inhibition of STMN1 in treatments of cancer has been the focus of interest for many researchers (Sucharita et al., 2006, Akhtar et al., 2013). Small interfering RNAs (siRNAs) that stop gene expression and prevent the development and progression of many cancers became quite famous in cancer treatment. siRNAs serve as an RNA guide for proper micro RNA (miRNA) degradation (Kimberly et al., 2015). Although it has been demonstrated that STMN1 silencing reduced invasion in PCa cell line as in many cancer types, this is the first study to demonstrate the effects on the autophagy, apoptosis, and invasion genes expressions of STMN1 silencing transfection in PC-3 cell lines. Similar to other studies in the literature, we observed a positive relationship between increased tumor burden and overexpression of STMN1 immunohistochemically, in the present study (Mistry et al., 2005, Akhtar et al., 2013).

We examined the effects of siRNA-mediated STMN1 silencing on the expression of main autophagy, invasion, and apoptosis pathway-related genes (*BAK1*, *BCL2*, *CASP*, *CTSB*, *FGF8*, *MMP2*, *AMBRA1*, *ATG2-5*, *ULK1*, etc.). In the current study, although we observed that silencing of STMN1 with siRNA knockdown all of some invasion, autophagy, and apoptosis gene expressions in the PCa cell line, the most significant knockdown was in the invasion genes.

It is generally known that cancer develops as a result of an increase in cell proliferation and a decrease in apoptotic mechanisms. Cancer cells try to escape the cell death pathway by increasing antiapoptotic mechanisms or by inactivating proapoptotic mechanisms. Apoptosis mechanisms are activated through two different pathways called intrinsic and extrinsic. Both of these pathways activate a series of caspases, targeting and lysing key cellular proteins. Decreased functions of caspases constitute one of the escape mechanisms from apoptosis. Caspases (CASP) are classified according to their structural features as inflammatory CASPs such as CASP 1, 4, and 5, initiator CASPs such as CASP 2, 8, 9, 10, and effector CASPs such as CASP 3, 6, 7 (Bildik et al., 2018). In our study, we found that the levels of all of CASPs classes, which we expected to increase with siRNA-mediated STMN1 silencing, decreased. It was observed that patients with gastric cancer with CASP 3 expression had a better prognosis than those without; however, in this study CASP3 was unaffected by siRNA-mediated STMN1 silencing (Huang et al., 2018). Similarly, it was determined that there was a decrease in the levels of CASP8 in patients with breast cancer. In our study, we found that the levels of CASP8 decreased after siRNA-mediated STMN1silencing, which we expected to increase with siRNA-mediated STMN1 silencing (Aghababazadeh et al., 2017). It has been observed in studies that siRNA-mediated STMN1 silencing increases the effect of cytotoxic agents such as taxanes, working by affecting the tubules (Alli et al., 2021). Meng et al. showed that the efficacy of docetaxel, used in the treatment of gastric cancer, was significantly increased when used in combination with siRNA-mediated STMN1 silencing in both in vivo and in vitro (Meng et al., 2016). In this study, STMN1 silencing was determined to significantly increase caspase 3 protein expression, but not change microtubule-associated proteins 1A/1B light chain 3B protein expression (p < 0.001 and p = 0.32), respectively. Although PCR and western blot results were not compatible with each other, we thought that STMN1 was significantly effective in caspase the apoptotic pathway in PCa due to the increase of CASP3 after siRNA-mediated STMN1 silencing via western blot analysis. Wang et al. also showed that apoptosis increased via upregulated caspase genes especially CASP3 by knockdown of STMN1 in squamous cell carcinoma of head and neck, and gall bladder carcinoma (Wang et al., 2016). Again similarly, Wu and colleagues determined that combination paclitaxel and siRNA-mediated STMN1 silencing increased to tending of apoptosis in nasopharynx carcinoma cell lines (Wu et al., 2014). The inconsistency between PCR and western blot results in apoptosis-related genes suggests that the effect of STMN 1 silencing on apoptosis is not very clear. In addition, the pleiotropic nature of STMN1 may contribute to this situation. The direction of the oncogenesis is determined according to the direction of the wind. More detailed studies on this subject will illuminate the gray zones.

Autophagy acts bidirectionally in tumor cells. In the limited stage, it prevents distant metastasis by preventing necrosis and inflammation. In the advanced stage of cancer, it helps the disintegration of the EMT zone, and cancer cells stay adhere-reside or dormant in the new microenvironment. There is a strong relationship between the apoptotic and autophagy pathways. In the presence of any autophagy defects, cancer transformation is facilitated with adverse microenvironmental conditions; this promotes the survival, proliferation, and growth of cancer cells (Cristofani et al., 2014). Studies with animal models have shown that autophagy suppresses the growth of benign tumors but supports the progression of malignant tumors (Onorati et al., 2018). In the current study, *ATG2A*, *ATG5*, and *ULK1* autophagy-related genes are inhibited; as a result, autophagy was not markedly induced by siRNA-mediated STMN1 silencing.

Wang and colleagues showed that the invasion of PCa reduced by silencing of STMN1 in gallbladder cancer; however, they used miRNA 34a for STMN1 inhibition in their study (Wang et al., 2016). Similarly, invasion-related genes including *CLDN7*, *FGF8*, *HIF1A*, *HGF*, *MMP2-7* proteins were downregulated via siRNA-mediated STMN1 silencing in the present study. From these deductions, we suggest that STMN1 has the main role in the invasion of PCa.

*FGF8* has a role in many processes, including cell division, regulation of cell growth, and maturation. Overexpression of *FGF8* has been shown to increase tumor proliferation rate and angiogenesis (Mattila et al., 2007). Especially, it has been observed to be higher in hormonal-dependent (i.e. androgen-induced) prostate and breast cancers (Tanaka et al., 1995). *FGF8* has been associated more frequently in events with hematogenous spread and bone metastases in advanced stage PCa. In our study, we also determined that *FGF8* knockdown by STMN1 with siRNA. We concluded that STMN1 plays role in the dissemination of PCa via hematogenous spread.

Tight junctions are very important for the continuity of EMT. EMT loss is essential in the processing mechanism of certain epithelial cancers. CLDN family is likely the cornerstone for this barrier against invading tumoral processing. In a previous study, it was determined that *CLDN4* is down-regulated, *CLDN 2*, *3*, *5* upregulated, and *CLDN7* does not change in PCa (Malheiros et al., 2011). While overexpression *CLDN7* is associated with some cancers, it has been found knockdown of *CLDN7* in some cancers such as breast cancer and H. pylori-associated gastric cancers (Dahiya et al., 2011, Singh et al., 2015, Wroblewski et al., 2015). Malheiros and colleagues also deducted that *CLDN7* was upregulated by androgens in PCa, from in their study (Malheiros et al., 2011). Similarly, we saw that *CLDN7* can be silenced by siRNA-mediated STMN1 silencing in our study.

It has been known that *HIF1* is activated under hypoxic conjectures’ in tumor cells, but sometimes *HIF1A* activation can occur under oxygen-independent conditions. Cancer-specific alterations including genetic variations, mutations may cause this situation to occur (Luo et al., 2018).

AHIF1A pathway has also been implicated in the etiopathogenesis of mCRPCa. Tran and colleagues, in their study, showed that a prognostic relationship between AR, HIF1A, hypoxia, and progression status in PCa (Tran et al., 2020). We showed that *HIF1A* might be sustained by STMN1 with siRNA in the present study.

Hepatocyte growth factor (HGF) regulates cell growth, cell motility, morphogenesis, and angiogenesis via the tyrosine kinase pathway through a proto-oncogene c-Met receptor. Recent studies have shown that Wnt/β–catenin signaling and HGF/Met are activated in metastatic processing PCa, including mCRPCa (Aldahl et al., 2020). HGF is overexpressed and associated with poor prognosis, formation of bone metastases in advanced-stage PCa cases, and it has been associated with lymph node involvement in locally advanced PCa (Pisters et al., 1995). In this study, it was shown that *HGF* can be suppressed by STMN1 with siRNA.

Epithelial-mesenchyme transition (EMT) is an important barrier to the invasion of tumor cells and also plays a role in cell adhesion, cell-to-cell cross-talk, and cell differentiation. When these barriers are disrupted through such as serine proteases, threonine proteases, and matrix metalloproteinase (MMP), cell integrity is broken and metastases occur. Geng et al. showed that MMPs have important roles in the stages of tumor genesis and progression of the PCa (Geng et al., 2020)). The researchers determined that *MMP-2* expression detected in the presence of micrometastases in the bone marrow after radical prostatectomy indicated a higher Gleason score, shorter PFS, the presence of circulating PCa cells (Murray et al., 2020). Similarly, Tregunna and colleagues described that *MMP7* has prognostic value for survival and response to treatment chosen in PCa (Tregunna et al., 2020). Similar to the mentioned studies, we have seen that *MMP2-7* might be knockdown by STMN1 with siRNA in the invasion of the PCa and that invasive patterns can be managed in this way.

The present study has several limitations. Firstly, the number of patients diagnosed with PCa was limited. If we had more cases, we could talk about statistical significance in Cox regression analysis more comfortably. Secondly, we did not investigate other probable interweaving modifications of these genes and genetic variations that could occur along the cell lines. Thirdly, only one PCa cell line was used. Fourthly, examination of STMN1 was only conducted by immunohistochemistry, not blood samples.

In conclusion, it was determined that histopathological samples obtained from PCa patients were STMN1 immune reactive. It was observed that invasion genes were suppressed more than apoptosis, and autophagy-related genes by siRNA-mediated STMN silencing in the PCa cell line. Lower STMN1 is a favorable factor in terms of survival. The siRNA-mediated STMN1 silencing suppressed invasion of the PCa cell line. These findings indicated that siRNA-mediated STMN1 silencing might serve as a potential therapeutic target for PCa.

## Figures and Tables

**Table 1:** The relationship stathmin1 (STMN1) immunoreactivity histoscore of paraffin block prostate tissue specimens for each group compared with a control group.

Group	STMN1*	p-value
Control	0.48 ± 0.16	-
Grade 1	0.68 ± 0.35	0.610
Grade 2	1.18 ± 0.62	0.010
Grade 3	1.24 ± 0.72	0.017
Grade 4	1.39 ± 0.80	0.009
Grade 5	1.48 ± 0.87	0.008

* Histoscore (prevalance × severity):

* Mean value ± SD, One-way ANOVA used

**Table 2:** The relationship between clinical features and stathmin1 (STMN1) expression rates.

Variables	n, (%)	STMN1*	p-value
Age			0.030
<65 years	17 (18.9%)	0.73 ± 0.11	
≥65 years	73 (81.1%)	1.15 ± 0.75	
Stage			0.162
Local advanced	35 (46.7%)	1.07 ± 0.63	
Advanced	40 (53.3%)	1.31 ± 0.80	
Perineurol invasion			0.970
Positive	35 (46.7%)	1.23 ± 0.83	
Negative	40 (53.3%)	1.16 ± 0.64	
Angiolymphatic invasion			
Positive	31(41.3%)	1.31 ± 0.79	0.246
Negative	44(58.7%)	1.11 ± 0.68	
Risk group-Gleason Scoreπ			0.010
≥7	45 (60%)	1.37 ± 0.11	
<7	30(40%)	0.93 ± 0.10	
Status of progression			0.528
Present	23(30.7%)	1.23 ± 0.75	
Absent	52 (69.3%)	1.10 ± 0.69	
Location of metastases			0.045
Bone metastases	25 (33%)	1.27 ± 0.78	
Lung metastases	2 (2.6%)	1.35 ± 0.63	
Multiple visceral involvement	8 (10.6%)	1.86 ± 0.83	
Nodal involvement	5 (6.6%)	0.6 ± 0.273	

* mean ± SD,

π According to AJCC 8 ≥ 7: Very high, high, and unfavorable intermediate risk group, < 7: Very low, low, and favorable intermediate risk group.

**Table 3:** Analysis based on stathmin1 (STMN1) staining results.

Stathmin1				
	Case (n, %)	L-STMN1	H-STMN1	p*-*value
BPH (as a control group)	15 (100%)	15(100%)	-	
Case (n)	75 (100 %)	27 (36 %)	48 (64 %)	
		0.5 ± 0.13*	1.81 ± 1.63	0.001^a^
Age*	71.53 ± 6.83*	71.36 ± 6.61*	71.69 ± 7.09*	0.92#
<65 years	17 (18.9%)	10 ( 23.8%)	7 (14.6 %)	0.26ë
≥65 years	73(81.1%)	32 (76.2%)	41 (85.4%)	
Stage				0.50ë
Local advanced	35(46.7%)	14 (51.9 %)	21 (43.8 %)	
Advanced	40 (53.3%)	13 (48.1%)	27 (56.2%)	
Gleason Score				0.11ë
≥ 7	45 (60%)	13 (48.1%)	32 (66.7%)	
< 7	30 (40%)	14 (51.9%)	16 (33.3%)	
Progression				0.37ë
Present	23 (30.7%)	10 (37%)	13 (27.1%)	
Absent	52 (69.3%)	17 (63%)	35 (72.9%)	
Perineurol invasion				0.50ë
Positive	35 (46.7%)	14 (51.9%)	21 (43.8%)	
Negative	40 (53.3%)	13 (48.1%)	27 (56.2%)	
Angiolymphatic invasion				0.57ë
Positive	31 (41.3%)	10 (37%)	21 (43.8%)	
Negative	44 (58.7%)	17 (63%)	27 (56.2%)	

# Mann–Whitney U test used,

* Mean values,

a T test used,

ë Pearson chi-squared,

L: Low, H: High

**Table 4:** Cox proportional hazards for the predictor of survival.

Multivariate analysis			
Variable	*p-*value	HR	CI
Age (<65≥)	0.013	10.501	1.649–66.88
LDH value	0.046	1.004	1.000–1.008
Progression (+/−)	0.526	0.456	0.040–5.149
Gleason Score	0.177	5.112	0.479–54.530
STMN1	0.169	2.141	0.724–6.327
Pre-PSA	0.012	1.000	1.000–1.000

HR: Hazard ratio, CI: Confidence interval, LDH: Lactic dehydrogenase enzyme

Pre-PSA: Pretreatment prostate specific antigen, STMN1: Stathmin1

**Table 5:** mRNA fold change and *p*-value in the PC-3 cell line compared to the control group after stathmin1 (STMN1) siRNA transfection.

		Negative control		STMN1 siRNA	
Symbol	Name	mRNA fold chance	*p-*value	mRNA fold charge	*p-*value
APAF1	Apoptotic peptidase activating factor 1	1.815	0.083	1.223	0.458
BAK1	BCL2-antagonist/killer 1	1.404	0.245	0.901	0.682
BAX	BCL2-associated X protein	1.434	0.223	0.908	0.701
BCL2	B-cell CLL/lymphoma 2	1.035	0.898	0.664	0.177
CASP1	Caspase 1, apoptosis-related cysteine peptidase	0.877	0.615	*0.325* a b	*0.021*
CASP2	Caspase 2, apoptosis-related cysteine peptidase	0.986	0.953	0.521	0.069
CASP3	Caspase 3, apoptosis-related cysteine peptidase	1.266	0.394	1.347	0.292
CASP7	Caspase 7, apoptosis-related cysteine peptidase	0.914	0.727	*0.476* a	*0.046*
CASP8	Caspase 8, apoptosis-related cysteine peptidase	0.865	0.581	*0.359* a b	*0.025*
DFFA	DNA fragmentation factor, 45kDa, alpha polypeptide	0.732	0.277	0.607	0.121
ADAMTS1	ADAM metallopeptidase with thrombospondin type 1 motif, 1	1.007	0.983	0.674	0.188
CD44	CD44 molecule (Indian blood group)	0.933	0.786	0.570	0.095
CDH1	Cadherin 1, type 1, E-cadherin (epithelial)	0.529	0.073	0.674	0.188
CDH2	Cadherin 2, type 1, N-cadherin (neuronal)	0.986	0.953	1.240	0.430
CDH6	Cadherin 6, type 2, K-cadherin (fetal kidney)	0.779	0.368	0.540	0.078
CDH11	Cadherin 11, type 2, OB-cadherin (osteoblast)	0.946	0.827	0.603	0.118
CLDN7	Claudin 7	0.824	0.471	*0.493* a	*0.049*
CSF3	Colony stimulating factor 3 (granulocyte)	0.693	0.216	0.591	0.109
CTSB	Cathepsin B	0.847	0.532	0.779	0.362
CTSD	Cathepsin D	1.705	0.106	*5.242* a b	*0.009*
FGF8	Fibroblast growth factor 8 (androgen-induced)	0.629	0.142	*0.339* a	*0.022*
HGF	Hepatocyte growth factor (hepapoietin A; scatter factor)	0.732	0.277	*0.392* a	*0.031*
HIF1A	Hypoxia inducible factor 1, alpha subunit	0.514	0.067	*0.454* a	*0.045*
MMP1	Matrix metallopeptidase 1 (interstitial collagenase)	0.889	0.651	0.669	0.182
MMP2	Matrix metallopeptidase 2 (gelatinase A)	0.717	0.252	*0.339* a b	*0.022*
MMP7	Matrix metallopeptidase 7 (matrilysin, uterine)	0.747	0.305	*0.314* a b	*0.019*
MMP10	Matrix metallopeptidase 10 (stromelysin 2)	1.301	0.347	1.231	0.444
MMP11	Matrix metallopeptidase 11 (stromelysin 3)	0.595	0.114	1.028	0.922
MMP13	Matrix metallopeptidase 13 (collagenase 3)	0.986	0.953	0.779	0.362
MMP14	Matrix metallopeptidase 14 (membrane-inserted)	0.717	0.252	1.165	0.568
TGFB1	Transforming growth factor, beta 1	0.551	0.085	1.064	0.816
TIMP2	TIMP metallopeptidase inhibitor 2	0.637	0.151	0.563	0.090
AMBRA1	Autophagy/beclin-1 regulator 1	0.674	0.191	1.347	0.292
ATG2A	ATG2 autophagy related 2 homolog A (S. cerevisiae)	0.732	0.277	*0.444* a	*0.042*
ATG3	ATG3 autophagy related 3 homolog (S. cerevisiae)	0.774	0.356	0.747	0.299
ATG5	ATG5 autophagy related 5 homolog (S. cerevisiae)	0.660	0.175	*0.359* a	*0.025*
ATG7	ATG7 autophagy related 7 homolog (S. cerevisiae)	0.835	0.501	1.257	0.403
ATG10	ATG10 autophagy related 10 homolog (S. cerevisiae)	0.607	0.124	0.664	0.177
MAP1LC3B	Microtubule-associated protein 1 light chain 3 beta	0.824	0.471	0.637	0.148
ULK1	Unc-51-like kinase 1 (C. elegans)	0.563	0.092	*0.135* a b	*0.008*
UVRAG	UV radiation resistance associated gene	1.007	0.983	0.616	0.128

Glyceraldehyde-3-phosphate dehydrogenase (GAPDH) was used as a housekeeping gene for the mRNA expression analysis.

a p < 0.05 versus control group.

b p < 0.05 versus negative control group.

## References

[b1-turkjbiol-46-3-239] Aghababazadeh M Dorraki N Javan FA Fattahi AS Gharib M 2017 Downregulation of Caspase 8 in a group of Iranian breast cancer patients - A pilot study Journal of the Egyptian Nati-onal Cancer Institute 29 4 191 195 10.1016/j.jnci.2017.10.001 29233452

[b2-turkjbiol-46-3-239] Alli E Bash-Babula J Jin-Ming Y Hait WN 2021 Effect of stathmin on the sensitivity to anti-microtubule drugs in human breast cancer Cancer Research 62 23 6864 6869 12460900

[b3-turkjbiol-46-3-239] Aldahl J Mi J Pineda A Kim WK Olson A 2020 Aberrant activation of hepatocyte growth factor/MET signaling promotes-catenin–mediated prostatic tumorigenesis Journal of Bio-logical Chemistry 295 2 631 644 10.1074/jbc.RA119.011137 PMC6956540 31819003

[b4-turkjbiol-46-3-239] Akhtar J Wang Z Zhang ZP Bi MM 2013 Lentiviral-mediated RNA interference targeting stathmin1 gene in human gastric cancer cells inhibits proliferation in vitro and tumor growth in vi-vo Journal of Translational Medicine 11 212 10.1186/1479-5876-11-212 24040910 PMC3848762

[b5-turkjbiol-46-3-239] Armstrong AJ 2018 Updates in advanced prostate cancer 2018 Prostate Cancer Prostatic Disea-ses 21 4 449 450 10.1038/s41391-018-0100-7 30279577

[b6-turkjbiol-46-3-239] Bildik A Bayar I 2018 Inhibition of apopitotic pathways in cancer Turkiye Klinikleri Journal of Veterinary Sciences 9 2 42 51

[b7-turkjbiol-46-3-239] Chen Z Wang T Cai L Su C Zhong B 2012 Clinicopathological significance of non-small cell lung cancer with high prevalence of Oct-4 tumor cells Journal of Experimental & Clinical Cancer Research 31 1 10 10.1186/1756-9966-31-10 22300949 PMC3287152

[b8-turkjbiol-46-3-239] Cristofani R Marelli MM Cicardi ME Fontana F Marzagalli M 2018 Dual role of autop-hagy on docetaxel-sensitivity in prostate cancer cells Cell Death & Disease 9 9 889 10.1038/s41419-018-0866-5 30166521 PMC6117300

[b9-turkjbiol-46-3-239] Dahiya N Becker KG Wood WH 3RD Zhang Y Morin PJ 2011 Claudin-7 is frequently ove-rexpressed in ovarian cancer and promotes invasion PLoS One 6 7 e22119 10.1371/journal.pone.0022119 21789222 PMC3137611

[b10-turkjbiol-46-3-239] Friedrich B Gronberg H Landstrom M Gullberg M Bergh A 1995 Differentiation-stage speci-fic expression of oncoprotein 18 in human and rat prostatic adenocarcinoma Prostate 27 2 102 109 10.1002/pros.2990270207 7638082

[b11-turkjbiol-46-3-239] Geng X Chen C Huang Y Hou J 2020 The prognostic value and potential mechanism of Mat-rix Metalloproteinases among Prostate Cancer International Journal of Medical Sciences 17 11 1550 1560 10.7150/ijms.46780 32669958 PMC7359399

[b12-turkjbiol-46-3-239] Ghosh R Gu G Tillman E Yuan J Wang Y 2007 Increased expression and differential phosphorylation of stathmin may promote prostate cancer progression Prostate 67 10 1038 1052 10.1002/pros.20601 17455228

[b13-turkjbiol-46-3-239] Golouh R Cufer T Sadikov A Nussdorfer P Usher PA 2008 The prognostic value of Stathmin-1, S100A2, and SYK proteins in ER-positive primary breast cancer patients treated with adjuvant tamoxifen monotherapy: an immunohistochemical study Breast Cancer Research and Tre-atment 110 2 317 326 10.1007/s10549-007-9724-3 17874182

[b14-turkjbiol-46-3-239] Howell B Larsson N Gullberg M Cassimeris L 1999 Dissociation of the tubulin-sequestering and microtubule catastrophe-promoting activities of oncoprotein 18/stathmin Molecular Biology of the Cell 10 1 105 118 10.1091/mbc.10.1.105 9880330 PMC25157

[b15-turkjbiol-46-3-239] Hu D Jıang L Luo S Zhao X Hu H 2020 Development of on autophagy-related gene expression signature for prognosis prediction in prostate cancer patients Journal of Translational Medicine 18 1 160 10.1186/s12967-020-02323-x 32264916 PMC7137440

[b16-turkjbiol-46-3-239] Huang KH Fang WL Li AFY Liang PH Wu CW 2018 Caspase-3, a key apoptotic pro-tein, as a prognostic marker in gastric cancer after curative surgery International Journal of Surgery 52 258 263 10.1016/j.ijsu.2018.02.055 29501797

[b17-turkjbiol-46-3-239] Luo Y Li M Zuo X Basourakos SP Zhang J 2018 β-catenin nuclear translocation induced by HIF-1α overexpression leads to the radioresistance of prostate cancer International Journal of Oncology 52 6 1827 1840 10.3892/ijo.2018.4368 29658569 PMC5919719

[b18-turkjbiol-46-3-239] Kimberly AB Yan YY Dominic CHN 2015 Loss of miR-223 and JNK Signaling Contri-bute to Elevated Stathmin in Malignant Pleural Mesothelioma Molecular Cancer Research 13 7 1106 1118 10.1158/1541-7786.MCR-14-0442 25824152

[b19-turkjbiol-46-3-239] Mattila MM Härkönen PL 2007 Role of fibroblast growth factor 8 in growth and progression of hormonal cancer Cytokine & Growth Factor Reviews 18 3–4 57 66 10.1016/j.cytogfr.2007.04.010 17512240

[b20-turkjbiol-46-3-239] Malheiros CCC Lourenco SV Fonseca FP Soares FA 2011 Claudin expression is dysregulated in prostate adenocarcinomas but does not correlate with main clinicopathological parameters Pathology 43 2 143 148 10.1097/PAT.0b013e3283428099 21233676

[b21-turkjbiol-46-3-239] Meng ZJ Tao K 2016 Enhancement of Chemosensitivity by Stathmin-1 Silencing in Gastric Cancer Cells In Situ and In Vivo Oncology Research 23 1–2 35 41 10.3727/096504015X14452563486057 26802649 PMC7842403

[b22-turkjbiol-46-3-239] Mistry SJ Bank A Atweh GF 2005 Targeting stathmin in prostate cancer Molecular Cancer Therapeutics 4 12 1821 1829 10.1158/1535-7163.MCT-05-0215 16373697

[b23-turkjbiol-46-3-239] Murillo-Garzón V Kypta R 2017 WNT signalling in prostate cancer Nature Reviews Urology 14 11 683 696 10.1038/nrurol.2017.144 28895566

[b24-turkjbiol-46-3-239] Murray NP Reyes E Salazar A Lopez MA Orrego S 2020 The expression of matrix-metalloproteinase-2 in bone marrow micro-metastasis is associated with the presence of circulating prostate cells and a worse prognosis in men treated with radical prostatectomy for prostate cancer Turkish Journal of Urology 146 3 186 195 10.5152/tud.2020.19219 PMC7219967 32401703

[b25-turkjbiol-46-3-239] NCCN Clinical Practice Guidelines in Oncology (NCCN Guidelines®). Version 2. 2021 Prostate Cancer Last accessed on February 17, 2021 https://www.nccn.org/professionals/physician_gls/pdf/prostate.pdf

[b26-turkjbiol-46-3-239] Nie W Xu MI-DIE Gan L Huang H Xiu Q 2015 Overexpression of stathmin 1 is a poor prognostic biomarker in non-small cell lung cancer Laboratory Investigation 95 1 56 64 10.1038/labinvest.2014.124 25384122

[b27-turkjbiol-46-3-239] Onorati AV Dyczynski M Ojha R Amaravadi RK 2018 Targeting autophagy in cancer Cancer 124 3307 3318 10.1002/cncr.31335 29671878 PMC6108917

[b28-turkjbiol-46-3-239] Pisters LL Troncoso P Zhau E Li W von Eschenbach AC 1995 c-met proto-oncogene expression in benign and malignant human prostate tissues Journal of Urology 154 1 293 298 7539865

[b29-turkjbiol-46-3-239] Sha J Han Q Chi C Zhu Y Pan J 2020 Upregulated KDM4B promotes prostate cancer cell proliferation by activating autophagy Journal of Cellular Physiology 235 3 2129 2138 10.1002/jcp.29117 31468537

[b30-turkjbiol-46-3-239] Siegel RL Miller KD Jemal A 2020 Cancer statistics, 2020 CA-A Cancer Journal for Clinici-ans 70 1 7 30 10.3322/caac.21590 31912902

[b31-turkjbiol-46-3-239] Singh AB Dhawan P 2015 Claudins and cancer: Fall of the soldiers entrusted to protect the gate and keep the barrier intact Seminars in Cell & Developmental Biology 42 58 65 10.1016/j.semcdb.2015.05.001 26025580

[b32-turkjbiol-46-3-239] Sucharita JM George FA 2006 Therapeutic interactions between stathmin inhibition and che-motherapeutic agents in prostate cancer Molecular Cancer Therapeutics 5 12 3248 357 10.1158/1535-7163.MCT-06-0227 17172428

[b33-turkjbiol-46-3-239] Tanaka A Miyamoto K Matsuo H Matsumoto K Yoshida H 1995 Human androgen-induced growth factor in prostate and breast cancer cells: its molecular cloning and growth properties FEBS Letters 363 3 226 230 10.1016/0014-5793 95 00324 3 7737407

[b34-turkjbiol-46-3-239] Tran MGB Bibby BAS Yang L Lo F Warren AY 2020 Independence of HIF1a and androgen signaling pathways in prostate cancer BMC Cancer 20 1 469 10.1186/s12885-020-06890-6 32450824 PMC7249645

[b35-turkjbiol-46-3-239] Tregunna R (2020) Serum MMP7 levels could guide metastatic therapy for prostate cancer Nature Reviews Urology 17 12 658 10.1038/s41585-020-00396-3 33173209

[b36-turkjbiol-46-3-239] Vlajnic T Bubendorf L 2021 Molecular pathology of prostate cancer: a practical approach Pathology 53 1 36 53 10.1016/j.pathol.2020.10.003 33234230

[b37-turkjbiol-46-3-239] Wang J Yao Y Ming Y Shen S Wu N 2016 Downregulation of stathmin 1 in human gallbladder carcinoma inhibits tumor growth in vitro and in vivo Scientific Reports 6 28833 10.1038/srep28833 27349455 PMC4923895

[b38-turkjbiol-46-3-239] Wu Y Tang M Wu Y Weng X Yang L 2014 A combination of paclitaxel and siRNA-mediated silencing of Stathmin inhibits growth and promotes apoptosis of nasopharyngeal carcinoma cells Cellular Oncology (Dordrecht) 37 1 53 67 10.1007/s13402-013-0163-3 24306928 PMC13004449

[b39-turkjbiol-46-3-239] Wroblewski LE Piazuelo MB Chaturvedi R Schumacher M Aihara E 2015 Helicobacter pylori targets cancer-associated apical-junctional constituents in gastroids and gastric epithelial cells Gut 64 5 720 730 10.1136/gutjnl-2014-307650 25123931 PMC4329117

[b40-turkjbiol-46-3-239] Williams K Ghosh R Giridhar PV Gu G Case T 2012 Inhibition of stathmin1 accelerates the metastatic process Cancer Research 72 20 5407 5417 10.1158/0008-5472.CAN-12-1158 22915755 PMC3543831

[b41-turkjbiol-46-3-239] Yan G Ru Y Wu K Yan F Wang Q 2018 GOLM1 promotes prostate cancer progression through activating PI3K-Aktm TOR signaling Prostate 78 3 166 177 10.1002/pros.23461 29181846

